# Resting Energy Expenditure in the Critically Ill and Healthy Elderly—A Retrospective Matched Cohort Study

**DOI:** 10.3390/nu15020303

**Published:** 2023-01-07

**Authors:** Matthias Lindner, Corinna Geisler, Kristina Rembarz, Lars Hummitzsch, David I. Radke, Dominik M. Schulte, Manfred J. Müller, Anja Bosy-Westphal, Gunnar Elke

**Affiliations:** 1 Department of Anesthesiology and Intensive Care Medicine, University Medical Center Schleswig-Holstein, Campus Kiel, Arnold-Heller-Str. 3 Haus 12, 24105 Kiel, Germany; 2 Institute of Diabetes and Clinical Metabolic Research, University Medical Center Schleswig-Holstein, Düsternbrooker Weg 17, 24105 Kiel, Germany; 3 Department of Human Nutrition and Food Science, Christian-Albrechts-University of Kiel, Düsternbrooker Weg 17, 24105 Kiel, Germany; 4 Division of Endocrinology, Diabetes and Clinical Nutrition, Department of Medicine I, University Medical Center Schleswig-Holstein, Campus Kiel, Arnold-Heller-Str. 3 Haus 12, 24105 Kiel, Germany

**Keywords:** resting energy expenditure, indirect calorimetry, elderly, medical nutrition therapy, critical care, calorie intake

## Abstract

The use of indirect calorimetry to measure resting energy expenditure (mREE) is widely recommended as opposed to calculating REE (cREE) by predictive equations (PE). The aim of this study was to compare mREE with cREE in critically ill, mechanically ventilated patients aged ≥ 75 years and a healthy control group matched by age, gender and body mass index. The primary outcome was the PE accuracy rate of mREE/cREE, derived using Bland Altman plots. Secondary analyses included linear regression analyses for determinants of intraindividual mREE/cREE differences in the critically ill and interindividual mREE differences in the matched healthy cohort. In this retrospective study, 90 critically ill patients (median age 80 years) and 58 matched healthy persons were included. Median mREE was significantly higher in the critically ill (1457 kcal/d) versus the healthy cohort (1351 kcal/d), with low PE accuracy rates (21% to 49%). Independent predictors of mREE/cREE differences in the critically ill were body temperature, heart rate, FiO_2_, hematocrit, serum sodium and urea. Body temperature, respiratory rate, and FiO_2_ were independent predictors of interindividual mREE differences (critically ill versus healthy control). In conclusion, the commonly used PE in the elderly critically ill are inaccurate. Respiratory, metabolic and energy homeostasis variables may explain intraindividual mREE/cREE as well as interindividual mREE differences.

## 1. Introduction

Elderly patients (defined by age ≥ 75 years) represent a growing proportion of intensive care unit (ICU) patients, with a steady increase in the mean age of the general ICU population over the past years [1,2]. A significant proportion of this cohort is even more elderly (≥80 years), with an annual increase reported of >5% [3]. Furthermore, during the first waves of the SARS-CoV-2 pandemic, elderly individuals were hospitalized more frequently compared to younger patients [4]. Among the elderly, the prevalence of malnutrition is reported to be high, ranging from 38% to 78%. This contributes to frailty, immobility, reduced muscle and fat-free mass, cognitive impairment, immunosuppression and impaired wound healing [5,6,7]. In addition, age-dependent decline in cellular metabolic activity and fat-free body mass is associated with a reduction in oxidative capacity, leading to lower resting energy expenditure (REE) as opposed to younger individuals [8,9,10]. Current nutrition guidelines for adult critically ill patients do not include specific recommendations for the elderly patient cohort [11,12]. Irrespective of age, these guidelines uniformly recommend the measurement of resting energy expenditure (REE) with indirect calorimetry (IC) in order to more precisely set the caloric target [11,12]. However, REE is still commonly estimated using predictive equations (PE) as IC is not available in the majority of ICUs. Predictive equations can be schematically divided into three groups: first, simplified PE using only one static variable (e.g., body weight); second, PE containing more than one static variable; and third, PE with a combination of static and dynamic variables (e.g., body temperature, heart or respiratory rate). Correction factors are frequently used in all types of formulas accounting for disease-dependent change in REE. In comparison to mREE, the PE of all these groups have been consistently shown to be inaccurate, resulting in frequent REE over- or underestimation [13,14,15,16,17]. Although various equations have been proposed for use in older critically ill adults, no PE specifically validated for this cohort yet exists. In one retrospective study, Harris–Benedict equation with correction factor was shown to be most accurate in critically ill patients with a mean age of 78 years [18].

The aim of our study was to compare (a) mREE with cREE, estimated by established predictive equations in critically ill, mechanically ventilated surgical patients aged ≥ 75 years and (b) to explore the physiological variables associated with differences between mREE and cREE, and between mREE of critically ill elderly and matched healthy controls.

## 2. Materials and Methods

### 2.1. Study Design and Setting

We conducted a retrospective cohort study at the Department of Anesthesiology and Intensive Care Medicine, University Medical Center Schleswig-Holstein, Campus Kiel and the Institute of Human Nutrition and Food Science, Christian-Albrechts-University Kiel, Kiel, Germany. The study was approved by the local ethics committee of the Christian-Albrechts-University, Kiel, Germany (File number: D 406/13). Due to the retrospective design and anonymous data analysis, the need for informed consent was waived by the ethics committee. Data of the critically ill patient cohort were obtained from the patient data management system of two intensive care units, and data of the matched healthy cohort were derived from the reference database of body composition and energy expenditure of the Institute of Human Nutrition and Food Science (File number: A 139/02).

### 2.2. Participants

#### 2.2.1. Critically Ill Patient Cohort

Inclusion criteria for the critically ill patient cohort were defined as follows: age ≥ 75 years admitted to the ICU, mechanical ventilation during the first 48 h with a fraction of inspired oxygen (F_i_O_2_) <0.6 and respiratory rate ≤30 per minute. Exclusion criteria were continuous hemofiltration, intermittent dialysis, inhaled NO therapy, bronchopleural fistula, respiratory or severe hemodynamic instability (defined as mean arterial pressure below 65 mmHg despite vasopressor therapy), chest drainage, and the administration of excess amounts of carbohydrates. The level of consciousness was documented using the Richmond agitation and sedation scale (RASS) [19]. All consecutive patients during a 24-month period were assessed for eligibility. Baseline patient characteristics, including medication, nutrition and physiologic variables, were recorded for the day of REE measurement along with data for acute physiology and chronic health evaluation II (APACHE II) [20], sequential organ failure assessment (SOFA) score [21], and nutrition risk in the critically ill patient (NUTRIC) score [22]. Indirect calorimetry was conducted with the open-circuit, side-stream M-CVOX IC device (Datex-Ohmeda, Helsinki, Finland). As per the clinical standard, a resting period with avoidance of physical activity of at least 30 min preceded all measurements. Before each measurement, the endotracheal tube cuff was checked in order to avoid a leak >10% ((inspiratory tidal volume [mL] − expiratory tidal volume [mL])/inspiratory tidal volume [mL] × 100)) and a 5-min warm up period with automated calibration was performed according to the manufacturer’s specifications. The measurement of REE was performed for 45 min after reaching equilibrium for at least five minutes, with a variation in gas exchange and a respiratory quotient of less than ±10%. All measurements were carried out with patients lying in supine position. Individual mREE was compared to cREE using established PE from Cerra et al. (ACCP) [23], Harris and Benedict (HarrisBenedict) [24], Ireton-Jones et al. (IretonJones) [25], Faisy et al. (FaisyFagon) [26], Müller et al. (Müller) [27], and Frankenfield et al. (PennState) [28]. The PE were chosen as representative of their derivation cohort (none: ACCP; healthy: HarrisBenedict, Müller; ICU patients: FaisyFagon, PennState), and the type and number of contained variables: simplified equations including one static variable (ACCP); equations including ≥1 static variable (HarrisBenedict, IretonJones, Müller); PE including ≥1 static and additional dynamic variables (FaisyFagon and PennState). If appropriate, formula-specific correction factors (e.g., stress, trauma, burns) were used.

#### 2.2.2. Matched Healthy Cohort

Eligibility criteria for the matched healthy subject’s cohort were as follows: healthy, (implying physically and functionally fully independent), not acutely ill, age 75 years or older, and known to be on long-term medication. All participants of the healthy subject cohort were derived from the reference database containing data from subjects enrolled in different investigations. The purpose of all investigations was identical (i.e., determination of energy expenditure, metabolic health and body composition). One study was a population-based family study with three generations and another study was focused on elderly people, and study populations are described in detail elsewhere [27,29,30,31]. Written and informed consent to participate in the respective studies was obtained from each subject. Medication intake was screened before inclusion for agents interfering with REE measurement (caffeine, nicotine, alcohol consumption, opioids, β-blocking agents, antipsychotics, sedatives, glucocorticoids, and growth and thyroid hormones) and participants were excluded when taking any substance of these medication classes. Activity levels were assessed in some but not all studies. The study participants were excluded if any acute diseases were reported during the screening procedure.

The criteria for the matching process were defined based on gender, age and BMI. Indirect calorimetry was performed using a ventilated canopy (CareFusion, Yorba Linda, California, United States of America; formerly: VIASYS healthcare, Würzburg, Germany). System calibration was performed according to the manufacturer’s instructions. Room temperature was maintained in the thermoneutral zone. Participants were advised to fast overnight (12 h) and to avoid physical activity before REE measurements. REE data were collected in the supine position for 45 min after reaching equilibrium for at least five minutes. Directly after the measurements, blood for laboratory analyses was drawn and physiological variables (heart rate, temperature, medication, systolic and diastolic arterial blood pressure) were collected in addition to baseline demographic data.

### 2.3. Statistical Analyses

The primary outcome was the accuracy rate of the assessed predictive equations expressed in percentage of the critically ill patient cohort. We defined accuracy a priori as a deviation of the intraindividual calculated REE (kJ/d; kcal/d) from the measured REE of less than ±10%. This corresponded to overestimation, defined as cREE >10% from mREE, and underestimation, defined as cREE >10% from mREE. Secondary outcomes were (a) correlation, precision, bias and limits of agreement between mREE and cREE in the ICU cohort and assessed using Bland–Altman plots, (b) independent predictors of intraindividual differences between mREE and cREE in the critically ill patient cohort, and (c) independent predictors of interindividual differences in mREE between critically ill patients and healthy controls.

The sample size calculation was performed based on retrospective data which was previously published [18]. With 98% power at an alpha level of α = 5%, the minimum required sample size for the effect size of 5/8 = 0.625 was calculated to be at least 44 (two-sided, one sample *t* test, G Power software, Düsseldorf, Germany). To detect a difference of 270 kcal/d between mREE and cREE (5% chance of error; β risk of 5%), we calculated a sample size of 90 patients in the critically ill patient cohort. Descriptive data are presented as mean and standard deviation (SD) or median and interquartile range (IQR), as appropriate. For comparison and correlation of mREE and cREE in the critically ill patient cohort, Wilcoxon and Mann–Whitney *U* test, non-parametric Spearman correlation and Bland–Altman analyses were used. The agreement of mREE and cREE was assessed by Bland–Altman analysis, with a calculation of the mean difference (bias) and limits of agreement (LOA) defined as the SD of the bias multiplied by 2 [32]. Mann–Whitney *U* test or Wilcoxon test were used for comparisons of mREE in the ICU cohort and healthy controls as appropriate. Univariate linear and stepwise forward linear regression analyses were used to identify variables associated with differences between mREE and cREE in the critically ill patient cohort, as well as the mREE between the critically ill patient cohort and the matched healthy control cohort. Statistical analyses were performed using SPSS Version 18 (IBM Software, Chicago, IL, USA) and GraphPad Prism Version 5 (GraphPad Software Inc., San Diego, CA, USA). For all analyses, we considered a *p* value of <0.05 as statistically significant.

## 3. Results

### 3.1. Study Flow and Patient Characteristics

**Figure 1:**
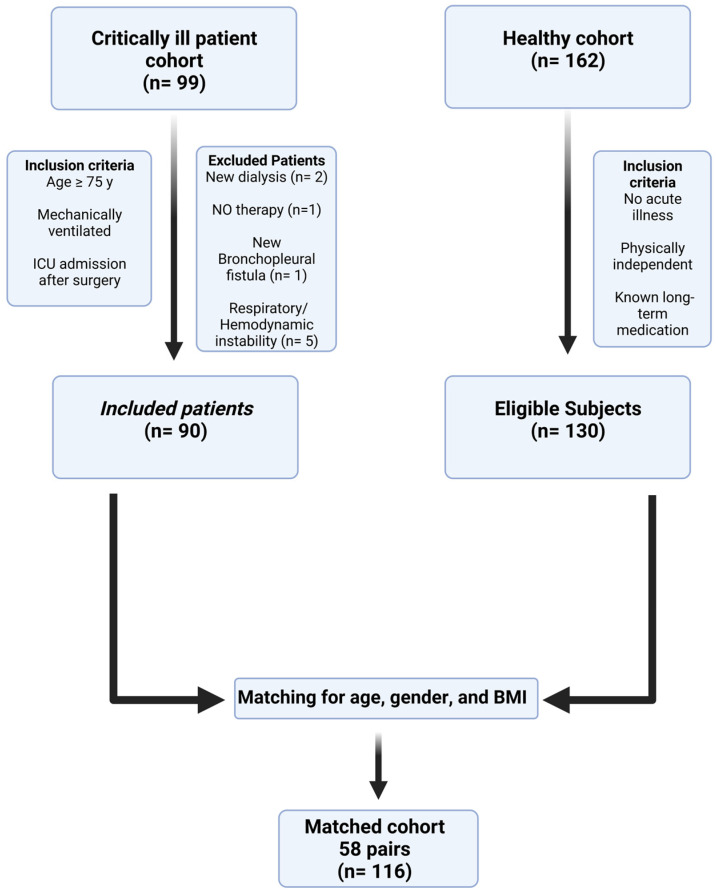
Study flow chart illustrating inclusion and matching process. The critically ill patient cohort comprised of N = 90 patients. For the matched cohort, N = 58 critically ill patients were matched by age, gender and BMI with subjects from the healthy cohort. Abbreviations. ICU: intensive care unit, NO: nitric oxide, BMI: body mass index.

Of 99 screened patients, 90 critically ill patients were finally included in the critically ill patient cohort with a median age of 80 years, a median APACHE II score of 19, a SOFA score of 7 and median NUTRIC score of 5. A total of 58 critically ill patients could be matched by age, gender and BMI with 58 healthy controls (Table 1, Figure 1). All physiologic variables, except for serum cholesterine levels, were significantly different between the matched pairs. In particular, participants of the matched healthy cohort had significantly higher systolic and diastolic blood pressure, and patients of the critically ill matched cohort had significantly higher heart rate and maximum body temperature.

### 3.2. Primary Outcome

ACCP had the lowest accuracy rate of 21.1%, according to N = 19 out of 90 patients. The accuracy rates of the other predictive equations were as follows: FaisyFagon (23.3%, N = 21/90), IretonJones (36.7%, N= 33/90), HarrisBenedict (40%, N = 36/90), Müller (41.1%, N = 37/90) and PennState (48.9%, N = 44/90). Median mREE in the critically ill patient cohort was 1475 kcal/d (interquartile range [IQR]: 1251–1892). Calculated REE by Harris–Benedict, Müller, FaisyFagon and PennState was significantly different from mREE (Table 2).

### 3.3. Secondary Outcomes

#### 3.3.1. mREE and cREE in the Critically Ill Patient Cohort

**Figure 2:**
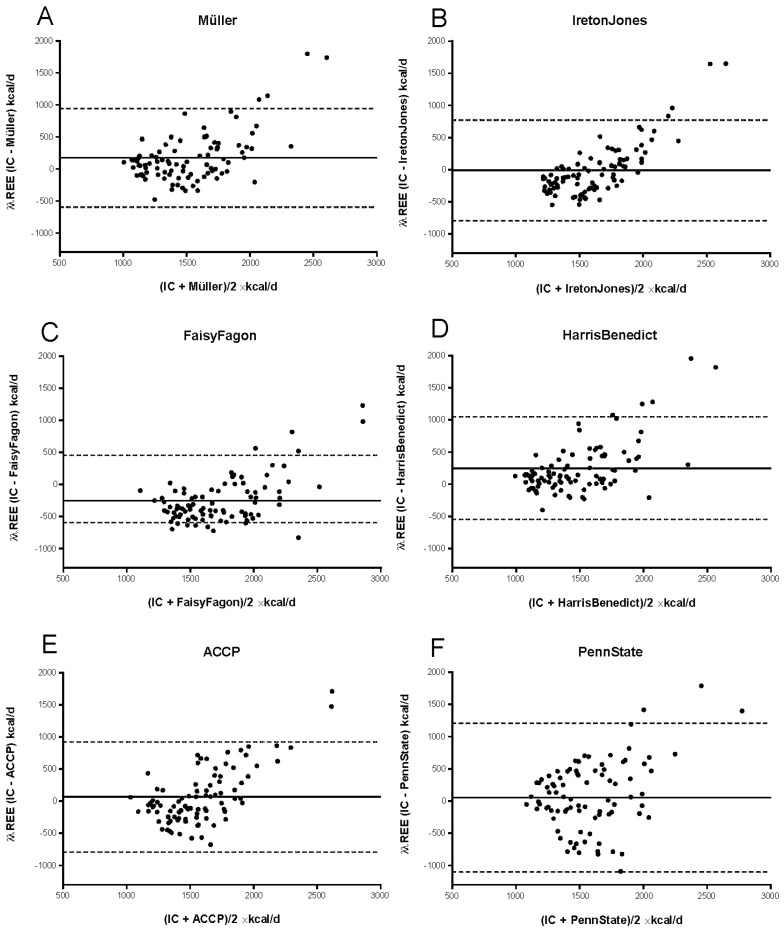
Bland–Altman analyses of measured and calculated resting energy expenditure in the critically ill patient cohort. The difference between the measured and calculated REE (*y* axis) is plotted against the mean of REE (*x* axis), with each data point corresponding to one patient for the following equations: (**A**) Müller, (**B**) Ireton-Jones and Jones, (**C**) FaisyFagon, (**D**) Harris and Benedict (**E**) ACCP, and (**F**) PennState. Abbreviations. REE: resting energy expenditure, kcal: kilocalories, d: day.

In summary, Bland–Altman plots revealed a systematic overestimation of mREE with Müller, Ireton-Jones, ACCP, and HarrisBenedict equations, whereas FaisyFagon and PennState were associated with both an over- and underestimation of mREE (Figure 2).

We found variations in bias, with overall wide limits of agreement in all six PE. Bias was lowest with IretonJones and highest with FaisyFagon and HarrisBenedict equations. cREE using IretonJones correlated best with mREE (r_s_: 0.615; y = 1.0261 × −1673.2), displaying no systematic difference (mean bias: −12.1 kcal/d), but a lack in precision (SD: 399.1 kcal/d) and wide limits of agreement (−794.5 kcal to 770.2 kcal/d). In contrast, FaisyFagon correlated poorly with mREE (r_s_: 0.318; y = 0.5765 × −1251.4), was systematically different (mean bias: −249.6 kcal/d), imprecise (SD of bias: 360.2 kcal/d), and had wide limits of agreement (−955.6 kcal/d to 456.5 kcal/d), as was PennState (mean bias: 56 kcal/d; SD of bias: 587.2 kcal/d; limits of agreement: −1094.9 kcal/d to 1206.9 kcal/d).

#### 3.3.2. Predictors of Differences between mREE and cREE in the Critically Ill Patient Cohort

**Figure 3:**
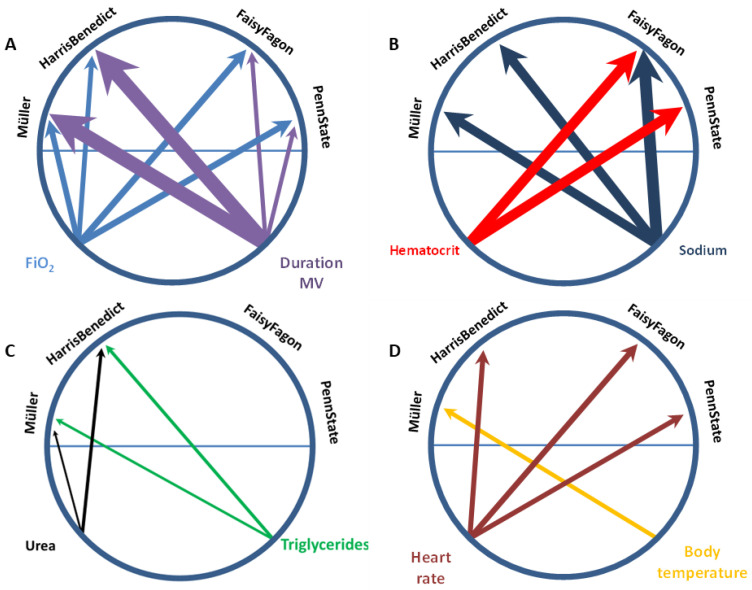
Variables independently associated with differences between estimated and measured resting energy expenditure in the critically ill patient cohort. Predictive equations developed for healthy persons are shown at the upper left side, and predictive equations developed for critically ill patients are shown on the upper right side of each panel. Panel (**A**) depicts respiratory (blue and purple), Panel (**B**,**C**) laboratory (red, dark blue, green, black), and Panel (**D**) circulatory and energy homeostasis variables (brown and yellow). The extent to which each variable explains differences between the two measurement methods (corresponding to change in R^2^ from Table 3) is represented by thinner or thicker arrows pointing to the respective predictive equation. For example, the duration of MV explains 26.6% (R^2^ of 0.226) of differences between measured resting energy expenditure and estimated resting energy expenditure using the equation of Müller (thicker purple arrow pointing to Müller), but only 7.9% (R^2^ of 0.079) of differences between measured resting energy expenditure and estimated resting energy expenditure using the PennState equation (thinner purple arrow pointing to PennState). Abbreviations: FiO_2_: Fraction of inspired oxygen; MV: mechanical ventilation; sodium: serum sodium concentration.

To assess which variables not included in the respective equations contribute to the observed differences between mREE and cREE, uni- and step-up forward linear regression models for the equations were used. Statistically significant differences between mREE and cREE were defined as the dependent variables. We identified body temperature, heart rate, FiO_2_, duration of mechanical ventilation, hematocrit, serum sodium concentration, and urea as independent predictors for intraindividual differences (Table 3). With regard to FaisyFagon and PennState, only urea levels were not independently associated with differences in REE. Conversely, for Müller and HarrisBenedict, only hematocrit was not significant. Figure 3 shows circular visualization plots for the relationship between independent predictors for REE differences and predictive equations used.

#### 3.3.3. Predictors for Differences in mREE between the Critically Ill Patient and Healthy Control Cohort

Measured REE was 1351 kcal/d (IQR: 1187–1503 kcal/d) in the matched healthy control cohort. When applying the REE equations developed for non-critically ill subjects and comparing it to the measured REE, we observed statistically significant differences for ACCP (1550 kcal/d, IQR: 1405–1749, *p* ≤ 0.001) and HarrisBenedict (1278 kcal/d, IQR: 1150–1388, *p* ≤ 0.001), but not for Müller (1453 kcal/d, IQR: 1167–162, *p* = 0.6696). In the healthy control cohort, ACCP had a poor correlation (r_s_: 0.6074, *p* ≤ 0.001) with a systematic difference (mean bias: 191.3 kcal/d), imprecision (SD of bias: 229.3 kcal/d), and wide limits of agreement (−258.2 to 640.7 kcal/d). HarrisBenedict had a similar correlation (r_s_: 0.6068, *p* ≤ 0.001) and systematic difference (−103.6 kcal/d), and was also imprecise (SD of bias: 198.2 kcal/d) with wide limits of agreement (−492 to 284.9 kcal/d). Müller had the best correlation (r_s_: 0.6797, *p* ≤ 0.001) and least systematic difference (mean bias: 8.1 kcal/d), but was also imprecise (SD of bias: 197.6 kcal/d) with wide limits of agreement (−379.1 to 395.4 kcal/d).

The measured REE in the healthy subjects was significantly when compared to the measured REE of the critically ill patient cohort (1457 kcal/d [IQR: 1247–1876 kcal/d]; *p* = 0.008), also when adjusted for BMI. Maximum body temperature (accounting for 6,6% of differences; Beta: −0.331; *p* = 0.008), respiratory rate (11.2%; Beta: −0.423; *p* ≤ 0.0001), and FiO_2_ (26.4%, Beta: −0.267; *p* = 0.022) were found to be independent predictors for the difference in mREE between the matched critically ill patient and healthy cohort.

## 4. Discussion

In this retrospective cohort study including 90 mechanically ventilated critically ill elderly patients (median age 80 years) and 58 healthy individuals matched by age, gender and BMI, mREE by indirect calorimetry was compared to cREE using six established predictive equations (ACCP, IretonJones, Müller, Harris–Benedict, PennState and FaisyFagon). Overall, the accuracy rates were low and did not exceed 48.9%, with the systematic over- and underestimation of REE and the lack of precision in all equations studied both in the critically ill and healthy control cohort. Parameters of respiratory function, metabolism and energy homeostasis not included in the respective equations were found to be independent predictors, an occurrence likely explaining the observed differences between intraindividual mREE and cREE. Measured REE was significantly lower in the matched healthy control and this could most likely explained by disease-specific patterns with maximum body temperature, respiratory rate and FiO_2_ found to be independent predictors for the difference in mREE. Differences in accuracy of the assessed PE may be due to the respective study population in which they were developed: whereas IretonJones, FaisyFagon and PennState were derived from critically ill patient cohorts, Müller, ACCP and HarrisBenedict were developed in healthy individuals. Accuracy of each PE may also depend on the use of dynamic and static variables: ACCP, with only static variables (body weight), has the lowest accuracy. Conversely, PennState uses multiple static and dynamic variables (weight, height, age, sex, minute volume ventilation and maximum body temperature) and performed better compared to the other assessed PE in the critically ill cohort. Likewise, in the matched healthy control cohort Müller, ACCP and HarrisBenedict correlated poorly with mREE and displayed systematic differences and imprecisions, with the PE by Müller being the most accurate. Accuracy rates of PE as compared to mREE varied in former studies of healthy elderly individuals but were consistently low, in line with our findings [33,34,35].

Despite the vast body of evidence showing that predictive equations are inferior for accurate determination of REE as opposed to the use of indirect calorimetry, data on REE in the increasing population of elderly patients in the ICU are still infrequent. In a retrospective study including 97 critically ill patients with a median age of 78 years, overall performance of the studied predictive equations was comparably low and the Harris–Benedict plus correction factor was shown to be most accurate [18]. In a prospective study including 194 non-critically ill patients at hospital admission and again three months after hospital discharge in 107 patients, best accuracy rates of the assessed equations were 40% at hospital admission and then 63% three months after discharge [33,36]. Equations combined with fat-free mass, height or illness factor performed slightly better. All PE had proportional bias, with overestimation of low REE values and underestimation of high REE values.

Factors contributing to REE changes in healthy persons and critically ill patients, including gender, physical activity, respiratory and endocrinological parameters and degree of systemic inflammation are well described [37,38]. Changes in body composition (decreased lean body mass) are considered to be the main drivers of the age-dependent decline in REE in elderly patients as compared to younger patients [39]. In a recent systematic review, Mtaweh and coworkers evaluated patient and clinical factors associated with energy expenditure in critically ill patients [40]. Ninety-five factors were significantly associated with energy expenditure, including minute volume, weight, age, the percentage of body surface area burn, sedation, post burn day, and caloric intake. Heart rate, fraction of inspired oxygen, respiratory rate, respiratory disease diagnosis, positive end-expiratory pressure, intensive care unit days, C-reactive protein, and size were not associated with energy expenditure. The authors concluded that a better understanding of underlying patient and clinical factors is important in the further development or adjustment of (existing) predictive equations. Accordingly, we tried to explain the intraindividual differences between cREE and mREE in our elderly cohort where body temperature, heart rate, FiO_2_, duration of mechanical ventilation, hematocrit, serum sodium concentration, and urea were identified as independent predictors, results which were partly in line with the findings of Mtaweh et al. Interestingly, hematocrit was the strongest predictor amongst the laboratory variables. Others reported that acute changes in hematocrit after transfusion of red blood cells or dilutional exchange transfusion may lead to changes in mREE [41,42]. Nevertheless, the mechanism and significance of mREE variations, which are dependent on acute hematocrit variations, still remain unclear. The plasma sodium concentration, involved in the maintenance of osmotic pressure, and regulation of water-homeostasis, amongst other physiological processes, was shown to be associated with differences in all equations except ACCP. However, experimental data show that an increase in plasma osmolality by the infusion of hypertonic sodium chloride solution can lead to an increase of VO_2_, and consecutive changes in plasma sodium concentration may therefore influence REE [38]. In summary, the incorporation of these variables into existing predictive equations may enhance their accuracy in the elderly critically ill. Each predictor had an impact ranging from small to modest at best, but in sum they explained up to over 70% of the observed differences.

Another study of 348 critically ill adult medical patients (mean age 65 years), comparing mREE to cREE with the Penn State, Swinamer, Ireton-Jones and ACCP equations, showed that age, BMI and gender were independent determinants of REE, with considerable variations between mREE and cREE [43]. Comparing interindividual mREE critically ill patients to healthy persons matched for age, gender and BMI, as identified in the study by Hölzel and coworkers, we further identified predictors that may be causally linked to the higher disease severity, i.e., maximum body temperature, respiratory rate and FiO_2_. A higher FiO_2_ may be the consequence of a reduction in oxygenation capacity in patients with pneumonia, with non-infective structural alterations of the lung parenchyma, such as atelectasis, or with changes in ventilation/perfusion ratio induced by positive pressure ventilation. Elevated heart rate may be caused by the experience of pain and agitation in the context of mixed or hyper-active forms of delirium, or by a thermoregulation center’s response to an infection, with both factors being associated with higher metabolic rates and therefore higher REE [44,45,46]. Again, to what extent the incorporation of these variables may enhance accuracy of existing or modified equations has to be proven in future prospective studies. In another study in critically ill children, however, no relationship was found between energy expenditure and clinical severity or with anthropometric, nutritional or biochemical status [47]. Differences in the accuracy of the assessed PE may be due to the respective study population in which they were developed: whereas IretonJones, FaisyFagon and PennState were derived from critically ill patient cohorts, Müller, ACCP and HarrisBenedict were developed in healthy individuals. The accuracy of each PE may also depend on the use of dynamic and static variables: ACCP with only static variables (body weight) had the lowest accuracy, whereas PennState uses multiple static and dynamic variables (weight, height, age, sex, minute volume ventilation and maximum body temperature) and performs better compared to the other assessed PE.

Our study has limitations to be addressed, including the retrospective design which inherently can only provide hypothesis-generating data. A main limitation is that potential bias may have arisen from REE measurements taken with two different methods, e.g., open-circuit/side-stream in the critically ill patients during mechanical ventilation vs. canopy hood devices in the spontaneously breathing healthy cohort. However, both IC devices used in this study are validated for a direct comparison, reducing systematic error introduced by potential confounding of inherent device-dependent measuring bias [48]. Body composition parameters were not available for the critically ill patient cohort and thus we were unable to further explore its influence on intra- and interindividual REE differences. Our study population was heterogeneous with respect to the underlying disease. However, on the other hand, it was also representative of a typical elderly ICU population. Participants of the matched healthy cohort were defined as healthy, however, blood pressure was observed to be in the higher-than-normal range. Furthermore, REE measurements were carried out only once, so no conclusions about longitudinal changes of REE can be drawn from this study. The strengths of our study include a standardized measurement protocol that was followed as per clinical or study standard operating procedures within the two databases, a well characterized cohort, and, to our best knowledge, the largest number of measured REE data in elderly ICU patients along with a respective matched healthy control.

In conclusion, our study shows that commonly used predictive equations for REE in elderly patients admitted to the ICU and in the healthy elderly are inaccurate and result in over- or underestimation of REE. Parameters of respiratory function, metabolism and energy homeostasis not included in the respective equations contribute to intraindividual differences in mREE and cREE. Measured REE was significantly lower in healthy elderly and intraindividual differences, a phenomenon explained by disease-dependent changes in maximum body temperature, respiratory rate and FiO_2_. According to current guideline recommendations, IC should also be preferably used in the elderly for most accurate REE determination and more personalized nutrition [11,12]. Further prospective studies are warranted to develop PE specific to the older adult in the ICU.

## Figures and Tables

**Table 1:** Baseline characteristics and physiologic data of the critically ill patients cohort (N = 90) and matched cohort (with values given for N = 58 healthy controls and N = 58 critically ill patients). All values are expressed as medians with 25% and 75% percentiles. Abbreviations. ACS: acute coronary syndrome; APACHE: acute physiological and chronic health evaluation; REE: resting energy expenditure; FiO_2_: fraction of inspired oxygen; SOFA: sequential organ failure assessment; NUTRIC: nutrition risk in critically ill; RASS: Richmond agitation and sedation scale; MV: mechanical ventilation; N: number of analyzed patients; mmHg: millimeters of mercury.

	Critically Ill Patients Cohort	Matched Cohort		*p*-Value
Anthropometrics	(N = 90)	Healthy Control (N = 58)	Critically Ill Patients (N = 58)	
Age, years	80 (77–84)	79 (76–81)	79 (76–82)	0.777
Gender, male, n (%)	44 (49). 46 w (51%)	25 m (43%). 33 f (57%)	25 m (43%). 33 f (57%)	0.139
Weight, kg	72 (60–88)	69 (60–80)	70 (60–81)	0.677
Height, days	168 (160–172)	165 (157–171)	165 (160–170)	0.206
BMI, kg/m^2^	25 (23–30)	25 (23–29)	25 (23–28)	0.738
Admission diagnosis				
Postoperative	25	-	18	n.a.
Perforation of a hollow organ				
(n)	14		9	
Pneumonia (n)	10		5	
Ischaemia of limbs or organ				
(n)	9		7	
Sepsis (n)	7		4	
Ileus (n)	6		3	
Bleeding (n)	6		5	
Trauma (n)	6		3	
ACS (n)	3		2	
Intracerebral bleeding (n)	1		1	
Seizures (n)	1		1	
Subdural hematoma (n)	1		0	
Vital Signs				
Heart rate, min^−1^	88 (76–103)	68 (60–72)	86 (74–103)	<0.00001
Systolic blood pressure, mmHg	121 (104–136)	150 (130–160)	122 (103–136)	<0.00001
Diastolic blood pressure, mmHg	53 (47–60)	90 (75–90)	54 (49–65)	<0.00001
Maximum body temperature, °C	37 (37–38)	36 (36–37)	38 (37–38)	<0.00001
Indirect Calorimetry				
Measured REE, kcal/d	1475 (1251–1892)	1351 (1187–1503)	1457 (1247–1876)	0.008
Respiratory Quotient, VCO_2_/VO_2_	0.76 (0.71–0.81)	0.82 (0.76–0.87)	0.76 (0.71–0.85)	0.016
Ventilatory parameters				
Days since start of MV	1 (1–2)	-	1 (1–2)	-
Days on MV	1 (1–2)	-	1 (1–2)	-
F_I_O_2_, %	40 (35–50)	-	40 (35–50)	-
Minute volume ventilation, L/min	8 (6–10)	-	8 (7–10)	-
Highest Respiratory rate, min^−1^	17 (9–23)	-	18 (9–27)	-
Partial pressure of O_2_, mmHg	108 (92–133)	-	104 (89–125)	-
Partial pressure of CO_2_, mmHg	46 (39–52)	-	44 (39–52)	-
Laboratory data				
Triglycerids, mg/dL	91 (53.3–134.5)	126.4 (87.7–166.0)	92.0 (50.0–134.0)	0.017
Cholesterine, mg/dL	85 (60.5–132.5)	188.7 (2.6–234.3)	86.0 (67.0–143.5)	0.083
Blood glucose, mg/dL	145 (119–127)	97 (84–106)	143 (118–173)	<0.00001
Thyrotropine, pg/mL	2.2 (0.9–3.7)	1.0 (0.8–1.4)	2.2 (1.0–3.8)	0.003
Unbound Tri-jodthyronine, pg/ml	1.7 (1.4–2.2)	3.0 (2.6–3.8)	1.7 (1.3–1.9)	0
Unbound Thyroxine, ng/dL	1.1 (1.0–1.4)	1.3 (1.1–1.6)	1.1 (0.9–1.2)	0.014
Creatinine, µmol/L	1.3 (0.9–1.8)	-	1.3 (0.9–1.7)	-
Urea, mmol/L	53.5 (33.0–81.5)	-	48.0 (27.0–80.0)	-
Hematocrit, %	30 (27.0–33.0)	-	30.0 (25.5–33.0)	-
Albumin, g/dL	2.1 (1.8–2.8)	-	2.2 (1.8–2.7)	-
Arterial pH	7.4 (7.3–7.4)	-	7.4 (7.3–7.4)	-
Base excess	−1.4 (−3.3–1.6)	-	−0.3 (−2.5–2.5)	-
Na^+^, mmol/L	139 (136.0–143.0)	-	140.0 (136.0–143.3)	-
K^+^, mmol/L	4.9 (4.3–5.2)	-	4.8 (4.3–5.1)	-
Thrombocytes, N × 10^9^/L	183 (115.5–268.3)	-	175.0 (109.8–238.5)	-
Bilirubine, µmol/L	0.6 (0.4–0.9)	-	0.6 (0.4–0.9)	-
Procalcitonin, µg/d	1.3 (0.3–3.8)	-	1.2 (0.3–3.4)	-
C-reaktive protein, mg/L	98.6 (46.9–205.5)	1.4 (1.1–2.4)	102.6 (51.1–182.3)	<0.00001
Leukocytes, N × 10^9^/L	11.9 (8.9–16.1)	-	11.5 (8.8–16.4)	-
Scores				
RASS, points	8 (7–9)	-	8 (7–9)	-
APACHE-II, points	19 (15–21)	-	18 (15–21)	-
SOFA, points	7 (4–8)	-	6 (4–8)	-
NUTRIC, points	5 (4–6)	-	5 (4–6)	-
Mortality, N (%)	48 (53)	0 (0)	31 (53)	<0.00001

**Table 2:** Calculated and measured median resting energy expenditure in the critically ill patient cohort. Significance of Δ mREE-cREE (kcal/d) was assessed using Wilcoxon test; differences are given for the whole cohort as the mean of the intraindividual differences between measured and estimated resting energy expenditure. Abbreviations: ACCP: REE calculated from Cerra et al.; FaisyFagon: REE calculated from Faisy et al.; HarrisBenedict: REE calculated from Harris and Benedict; IretonJones: REE calculated from IretonJones et al.; Müller: REE calculated from Müller et al.; kcal: kilocalories; d: day; Δ mREE-cREE (kcal/d): difference between measured and calculated resting energy expenditure expressed in kilocalories per day; IQR: interquartile range.

Predictive Equation	mREE, kcal/d	cREE, kcal/d	Intraindividual ΔmREE-cREE, kcal	*p* Value
ACCP	1475 [1251–1892]	1339 [1158–1574]	33 [−247–288]	0.641
IretonJones		1673 [1443–1773]	109 [−297–147]	0.085
FaisyFagon		1824 [1653–2080]	−353 [−486–96]	<0.0001
PennState		1483 [1244–1759]	54 [−105–248]	0.023
Müller		1471 [1191–1683]	105 [−82–334]	<0.0001
Harris–Benedict		1339 [1158–1574]	139 [23–407]	<0.0001

**Table 3:** Multivariate regression analysis for differences between mREE and cREE in the critically ill patient cohort. Abbreviations: ACCP: REE calculated from Cerra et al.; FaisyFagon: REE calculated from Faisy et al.; HarrisBenedict: REE calculated from Harris and Benedict; IretonJones: REE calculated from Ireton-Jones et al.; Müller: REE calculated from Müller et al.; FIO_2_: fraction of inspired oxygen; (log): logarithmic transformation; n: number of analyzed patients from the ICU cohort; PennState: REE calculated from Frankenfield; Mifflin: REE calculated from Mifflin et al.; REE: resting energy expenditure; *: Wilcoxon test.

Independent Variables		Dependent Variable: Δ mREE-cREE			
		FaisyFagon	PennState	Müller	Harris–Benedict
Heart rate (min^−1^) (log) (n = 90)	Change in R^2^	0.106	0.093	-	0.093
	Beta	0.383	0.418	-	0.322
	*p* value *	<0.0001	<0.0001	-	0.001
Max. body temperature (°C) (log) (n = 90)	Change in R^2^	-	-	0.095	-
	Beta	-	-	0.324	-
	*p* value *	-	-	0.001	-
Days since start of MV (days) (log) (n = 90)	Change in R^2^	0.103	0.079	0.267	0.266
	Beta	0.347	0.356	0.503	0.499
	*p* value *	0.001	0.001	<0.0001	<0.0001
FIO_2_ (%) (log) (n = 90)	Change in R^2^	0.119	0.119	0.107	0.09
	Beta	0.356	0.355	0.306	0.27
	*p* value *	0.001	0.001	0.003	0.009
Urea (mmol/L) (log) (n = 88)	Change in R^2^	-	-	0.047	0.061
	Beta	-	-	0.226	0.258
	*p* value *	-	-	0.025	0.011
Hematocrit (%) (log) (n = 88)	Change in R^2^	0.186	0.188	-	-
	Beta	−0.28	−0.26	-	-
	*p* value *	0.007	0.015	-	-
Na^+^ (mmol/L) (log) (n = 90)	Change in R^2^	0.217	0.083	0.169	0.175
	Beta	0.458	0.415	0.399	0.414
	*p* value *	0.000	<0.0001	<0.0001	<0.0001
R^2^ total		0.732	0.704	0.685	0.685

## Data Availability

All data analyzed in this study are available from the corresponding author on reasonable request.
